# ﻿Further clarification on *Androsacemollis* Hand.-Mazz. (Primulaceae), with a description of a new species of *Androsace*

**DOI:** 10.3897/phytokeys.212.94037

**Published:** 2022-11-03

**Authors:** Yuan Xu, Hai-Fei Yan, Gang Hao

**Affiliations:** 1 Key Laboratory of Plant Resources Conservation and Sustainable Utilization, South China Botanical Garden, Chinese Academy of Sciences, Guangzhou 510650, China Key Laboratory of Plant Resources Conservation and Sustainable Utilization, South China Botanical Garden, Chinese Academy of Sciences Guangzhou China; 2 Center of Conservation Biology, Core Botanical Gardens, Chinese Academy of Sciences, Guangzhou 510650, China Center of Conservation Biology, Core Botanical Gardens, Chinese Academy of Sciences Guangzhou China; 3 College of Life Sciences, South China Agricultural University, Guangzhou 510642, China South China Agricultural University Guangzhou China

**Keywords:** *Androsacechimingiana*, new taxon, Primulaceae, taxonomy, Yunnan

## Abstract

The syntypes (*H.R.E. Handel-Mazzetti 8896* and *H.R.E. Handel-Mazzetti 9280*) of *Androsacemollis* Hand.-Mazz. are identified as two separate taxa based on critical examinations of herbarium specimens and field investigation. While *H.R.E. Handel-Mazzetti 8896* has been designated as the lectotype of *A.mollis*, we describe the other taxon, represented by *H.R.E. Handel-Mazzetti 9280*, as *A.chimingiana* Y.Xu & G.Hao **sp. nov.** The new species is morphologically similar to *A.hookeriana* Klatt and *A.laxa* C.M.Hu & Y.C.Yang but can be easily differentiated by its white corolla and obovate bracts.

## ﻿Introduction

*Androsace* L. is a large genus of Primulaceae, which contains ca. 150 species (Smith and Lowe 1997). This genus is widely distributed in the temperate and arctic zones of the Northern Hemisphere, with the modern center of diversity in Pan-Himalaya, which harbors more than 50% of the species (Hu and Yang 1986; Hu and Kelso 1996; Xu et al. 2016; Chen 2019).

**Figure 1.**
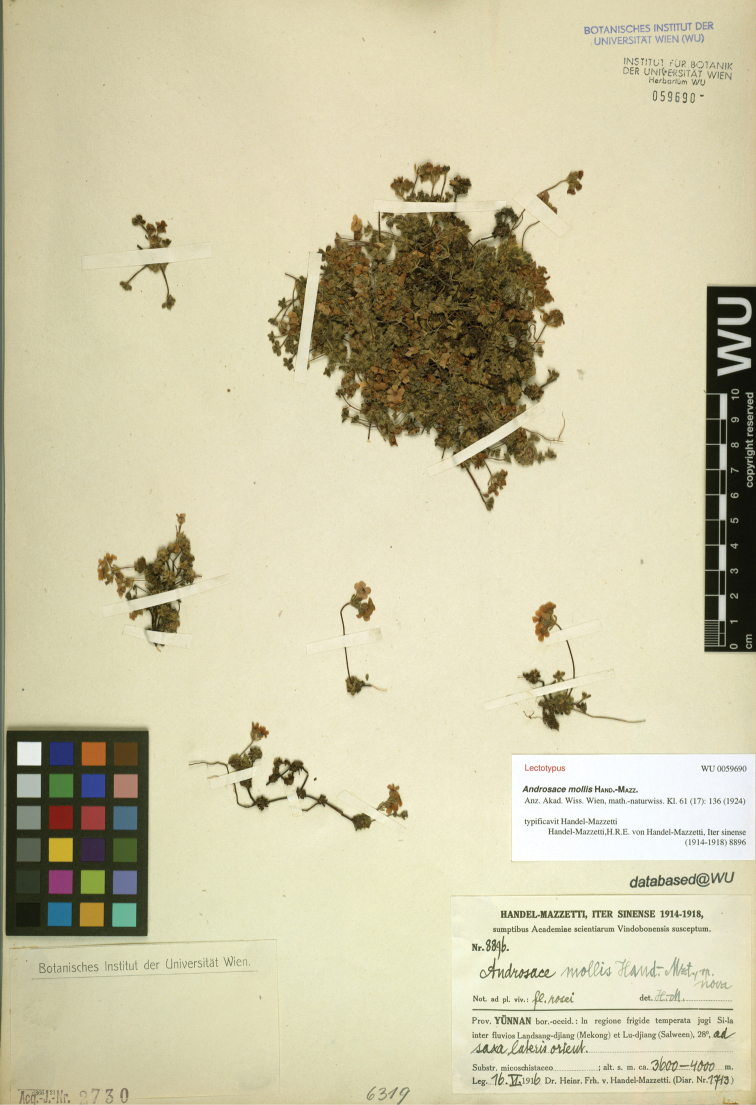
Lectotype sheet of *Androsacemollis* (*H.R.E. Handel-Mazzetti 8896*, WU barcode WU0059690, https://wu.jacq.org/WU0059690).

**Figure 2.**
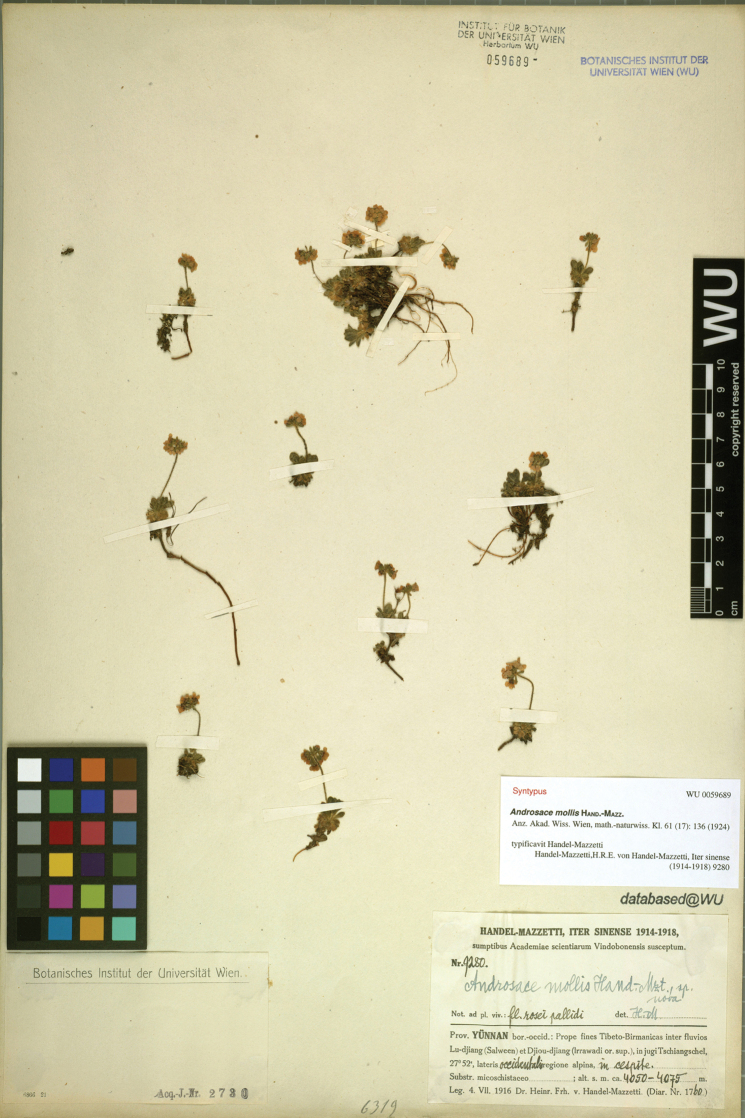
Paratype sheet of *Androsacechimingiana* (*H.R.E. Handel-Mazzetti 9280*, WU barcode WU0059689, https://wu.jacq.org/WU0059689).

When we revised the Chinese species of *Androsace*, the name *A.mollis* Hand.-Mazz. caught our attention. This name was described by Handel-Mazzetti (1924) based on two collections: *H.R.E. Handel-Mazzetti 8896* (Fig. 1) and *H.R.E. Handel-Mazzetti 9280* (Fig. 2) from Yunnan Province, China. However, in his later treatment of the *Androsace* species from China, he identified the herbarium specimens of *A.sublanata* Hand.-Mazz. (*J.M. Delavay 69* and *1038*) as his *A.mollis* (Handel-Mazzetti 1927; Xu et al. 2018). Then we checked the original description and the type specimens of *A.mollis*. According to the protologue, the leaves of *A.mollis* are scarcely dimorphous. But, the leaf morphology of the plants in *H.R.E.Handel-Mazzetti 8896* is homomorphic, while in *H.R.E. Handel-Mazzetti 9280* it is obviously dimorphous (Fig. 2): where the outer leaves are obovate while the inner leaves are spatulate and 1.5 to 2 times longer than the outer leaves. With our additional specimen examination and the field investigation in northwest Yunnan and southeast Xizang, it is confirmed that these two collections, cited in the protologue of *A.mollis*, represent two separate taxa. They can easily be differentiated by the morphology of leaf (homomorphic vs. obviously dimorphous) and bract (linear vs. obovate), the length of pedicel (same as the bract vs. 1.5 to 2 times of the bract), the depth of calyx splitting (less than 1/2 vs. 1/2 to 3/4) and the color of corolla (pink vs. white). Since Handel-Mazzetti (1936) designated *H.R.E. Handel-Mazzetti 8896* (Fig. 1) as the lectotype of *A.mollis* (corolla pink) under Art. 7.11 of the CIN (Turland et al. 2018), we describe the other taxon (corolla white) as a new species herein.

## ﻿Materials and methods

For morphological comparisons, both fresh and pressed specimens of the new species and its relatives were observed and measured. The main diagnostic characters (such as the indumentum of the plant, the shape of the leaf, calyx, calyx lobe and corolla lobe, the length of the shoot, scape and pedicel, and the color of the corolla) were measured and/or compared in the lab. Indumentum and other tiny morphological features were observed under a stereomicroscope. The herbarium specimens in Herbaria E, IBSC, K, KUN, LIV, P, SYS, SZ, US and WU were examined, with special attention on the specimens identified as *A.mollis* or cited by Handel-Mazzetti (1924, 1927). Field observations were conducted in 2012, 2013, 2015 and 2021 and focused on the variations of the morphology of leaf and bract, the depth of calyx splitting and the color of corolla. The field investigation covers all known distribution areas of the new species and *A.mollis*, including Dali Bai Autonomous Prefecture, Lijiang City, Diqing Tibetan Autonomous Prefecture and Nujiang Lisu Autonomous Prefecture of Yunnan, Zayu County of Xizang and MuLi County of Sichuan. The conservation status of *A.mollis* and the new species were assessed following the guidelines for using the IUCN Red List categories and criteria (IUCN Standards and Petitions Subcommittee 2022).

## ﻿Taxonomy treatment

### ﻿1. Androsace mollis

Taxon classificationPlantaeEricalesPrimulaceae

Hand.-Mazz.

D929148F-2786-526C-8863-F722E633B909

Fig. 1

Androsace mollis Hand.-Mazz. in Anzeiger der Akademie der Wissenschaften in Wien, Sitzung der Mathematisch-Naturwissenschaftliche Klasse 61: 136, 1924, *p.p. quoad specim*. Handel-Mazzetti 8896; Handel-Mazzetti in Notes from the Royal Botanic Garden, Edinburgh 15: 291, 1927, *p.p. quoad specim. F. K. Ward 6963*, *G. Forrest 7370*, *14263*, *14917*, *19307*, *J. F. Rock 8733*; C.M. Hu and Y.C. Yang in Acta Phytotaxonomica Sinica 24(3): 226, 1986, *p.p. quoad specim. G. Forrest 14263*, *H. R. E. Handel-Mazzetti 8896*, *J. F. Rock 21964*, *T. T. Yu 3703*; Y.C. Yang and R.F. Huang in Flora Reipublicae Popularis Sinicae vol. 59(1): 183, 1989, p.p. excl. syn. Androsacesarmentosavar.yunnanensis R. Knuth, *excl. specim.G. Forrest 1810*; C.M. Hu and S. Kelso in Flora of China vol. 15: 99, 1996, p.p. excl. syn. Androsacesarmentosavar.yunnanensis R. Knuth, *excl. speciminibus Sichuanensibus*; R.Z. Fang in Flora Yunnanica vol. 15: 392, 2003, *p.p. quoad specim.*Deqinense, Fugongense, Gongshanense.

#### Type.

China. Yunnan Province: Deqin County, in regione temperate jugi Si-la inter fluvios Landsang-djiang (Mekong) et Lu-djiang (Salween), 28°N, 3600–4000 m a.s.l., 16 June 1916, *H. R. E. Handel-Mazzetti 8896* (lectotype WU! barcode WU0059690; isolectotype E! barcode E00024874; K! barcode K000750338).

#### Description.

A perennial herb, laxly cespitose. ***Shoots*** densely villous and green when young, becoming glabrescent and dark reddish-brown, internodes 0.3–1.5 cm, with old petiole or leaf rosettes on nodes. ***Leaf rosettes*** 4–11 mm in diam. ***Leaves*** sessile, obovate-ligulate, unicostate, 3–7 mm × 1–2 mm, ciliate, densely covered with white multicellular hairs along the midrib on abaxial surface, and glabrescent on abaxial surface, apex obtuse to subacute. ***Scapes*** solitary, 0.5–3.5 cm long, sparsely white villous, carrying a terminal umbel with 1–6 flowers; bracts linear to linear-spatulate, unicostate, 2–4 mm × 0.5–1.5 mm, ciliate, densely covered with white multicellular hairs on distal 1/2 of abaxial surface, and sparsely villous on adaxial surface, base saccate, apex rounded; pedicels 2–4 mm long and can extend to 1 cm in fruiting, sparsely villous, dark reddish-brown. ***Calyx*** campanulate, 2–3 mm long, sparsely pubescent, parted less than 1/2 of its length; lobes ovate to ovate-oblong, ciliate, apex obtuse and reddish. ***Corolla*** pink with a yellow eye, limb 5–7 mm across, lobes obovate, apex entire or emarginate. ***Stamens*** ca. 1 mm, near corolla tube apex. ***Style*** ca. 2 mm. ***Capsules*** globose, glabrous, tawny, ca. 3 mm in diam, splitting to the base in valves. ***Blooming*** from June to August and fruiting from August to October.

#### Conservation status.

Based on our field investigation and specimen examination, *A.mollis* is widely distributed in northwest Yunnan, southeast Xizang and north Myanmar. Thus, the conservation status of *A.mollis* is assessed as Least Concern (LC) according to the guidelines for using the IUCN Red List categories and criteria (IUCN Standards and Petitions Subcommittee 2022).

#### Specimens examined.

**China. Yunnan**: *G. Forrest 7370* (E, K), *14263* (K), *14917* (K), *19307* (K); Deqin County *J.F. Rock 8733* (E, K, SYS barcode SYS00112510, US barcode 03124157), *23239* (K, KUN barcode 0211554, SYS barcode SYS00112511, US barcode 03124155), *K.M. Feng 5151* (KUN barcode 0211526, 0211527), *6580* (KUN barcode 0211529), *6644* (KUN barcode 0211530, 0211531), *T.T. Yu*, *22293* (KUN barcode 0211521, 0211522); Gongshan Derung and Nu Autonomous County *J.F. Rock 21964* (K, SYS barcode SYS00112512, US barcode 03124156), *T.T. Yu 19769* (KUN barcode 0211520), *22357* (KUN barcode 0211523, 0211524), *22783* (KUN barcode 0211525); Weixi Lisu Autonomous County *B. Xu Tsui-2038* (KUN). **Xizang**: Zayu County *Y. Xu Xu130135* (IBSC), *Y. Xu*, *S. Chen*, *J. Li Xu210933* (IBSC), *Y. Xu*, *T.J. Liu & G.H. Huang 150223* (IBSC), *J.F. Rock 22522* (K). **Upper Myanmar.***F. K. Ward 6963* (K).

#### Additional notes.

Although Handel-Mazzetti (1927) cited the specimens of *A.sublanata* (*J.M. Delavay 69* and *1038*) under his *A.mollis*, these two species are in fact quite different. The habit of *A.mollis* is laxly cespitose with obvious shoots while the leaf rosette of *A.sublanata* is solitary or 2–4 in small clumps without shoots. Moreover, *A.sublanata* is a somewhat larger species, with scapes 9–30 cm in length while the scapes of *A.mollis* are only 0.5–3.5 cm (Xu et al. 2018).

Hu and Kelso (1996) recorded that *A.mollis* is also distributed in western Sichuan. Then, Fang (2003) further indicated that *A.mollis* is distributed in MuLi County of Sichuan Province. We checked the Muli specimen (*Qinghai-Xizang Exped. 14477* KUN barcode 0211555) determined by Fang. It is *A.minor* (Hand.-Mazz.) C.M.Hu & Y.C.Yang instead of *A.mollis*. Based on our field investigation and herbarium specimen examination, the distribution areas of *A.mollis* only include northwestern Yunnan and southeastern Xizang of China and Northern Myanmar.

### ﻿2. Androsace chimingiana

Taxon classificationPlantaeEricalesPrimulaceae

Y.Xu & G.Hao sp. nov.

794FD8F4-6DEC-525E-8754-7E95940F223F

urn:lsid:ipni.org:names:77307629-1

Figs 2 , 3 , 4

Androsace mollis auct. non. Hand.-Mazz., Handel-Mazzetti in Anzeiger der Akademie der Wissenschaften in Wien, Sitzung der Mathematisch-Naturwissenschaftliche Klasse 61: 136, 1924, *p.p. quoad specim*. *Handel-Mazzetti 9280*.

#### Type.

China. Yunnan Province: Dali City, Cangshan, 25°40'N, 100°05'E, alt. 3830 m, 25 Jun. 2013 (fl.), *H.F. Yan*, *Y. Xu & S. Yuan Y2013045* (holotype IBSC!, isotype IBSC!).

#### Diagnosis.

*Androsacechimingiana* is similar to *A.hookeriana* Klatt and *A.laxa* C.M.Hu & Y.C.Yang, but differs in its white corolla with a yellow eye, obovate bracts, and campanulate calyx which is parted to 1/2–3/4 of its length.

#### Description.

A perennial herb, laxly cespitose. ***Shoots*** densely villous and green when young, becoming glabrescent and dark reddish-brown, internodes 0.5–3 cm, with old petiole or leaf single or rosettes on nodes. ***Leaves*** dimorphic; outer leaves sessile, obovate, unicostate, 3–8 mm × 1.5–2.5 mm, ciliate, densely covered with white multicellular hairs on abaxial surface, and sparsely villous on adaxial surface; inner leaves spatulate, unicostate, 8–14 mm × 2–5 mm, petiole indistinct to 1/2 as long as leaf blade, leaf blade elliptic to obovate, sparsely covered with white multicellular hairs on abaxial surface, and glabrescent on adaxial surface, margin spreading villous, more densely so at apex, apex obtuse to subrounded. ***Scapes*** 2.5–5.5 cm long, densely spreading white villous, carrying a terminal umbel with 4–8 flowers; bracts obovate, unicostate, 3–6 mm × 1–3 mm, ciliate, densely covered with white multicellular hairs on abaxial surface, and sparsely villous on adaxial surface; pedicels 5–8 mm long, sparsely villous, dark reddish-brown. ***Calyx*** campanulate, 2–3 mm long, sparsely pubescent, parted to 1/2–3/4 of its length; lobes narrowly ovate, ciliate, apex obtuse, margin reddish. ***Corolla*** white with a yellow eye, limb ca. 5 mm across, lobes obovate, apex subrounded or emarginate. ***Stamens*** 0.8–1 mm, near corolla tube apex. ***Style*** ca. 2 mm. ***Capsules*** ellipsoidal, glabrous, tawny, ca. 3 mm long, splitting to the base in valves.

#### Phenology.

*Androsacechimingiana* was observed blooming from May to July and fruiting from July to August.

#### Etymology.

The species name honors the Chinese botanist Prof. Chi-Ming Hu, who has made outstanding contributions to the taxonomy of Primulaceae.

#### Distribution and habitat.

Based on the collection records over the past 138 years (since 1884, *J.M. Delavay 1037*, P) as well as our recent field investigation, the new species is narrowly distributed in Cangshan Mountain (Dali City and Yangbi Yi Autonomous County) and the north part of Gaoligong Mountains (Gongshan Derung and Nu Autonomous County) in Nujiang River and Dulong River divide. However, *A.mollis* has a broader distribution range. It is distributed in the north part of Biluo Snow Mountain (*H.R.E. Handel-Mazzetti 8896* E, K, WU; *J.F. Rock 8733* E, K, SYS, US; *K.M. Feng 6580*, *6644* KUN; *T.T. Yu 22293* KUN) and west of Meri Snow Mountain (*J.F. Rock 23239* K, KUN, SYS, US) in Lancang River and Nujiang River divide and from the north part of Gaoligong Mountains in Nujiang River and Dulong River divide (*J.F. Rock 21964* K, SYS, US) to the southeast Xizang (*J.F. Rock 22522* K) and Upper Burma (*F.K. Ward 6963* K). Both the new species and *A.mollis* are distributed in the north part of Gaoligong Mountains. But no sympatric populations have been observed. The distribution area of *A.mollis* is more northerly than the new species. The habitats of these two species are also different. The new species grows under fir forest or *Rhododendron* thickets at 3000–4000 m a.s.l., while *A.mollis* grows on alpine meadow at 3800–4400 m a.s.l.

**Figure 3.**
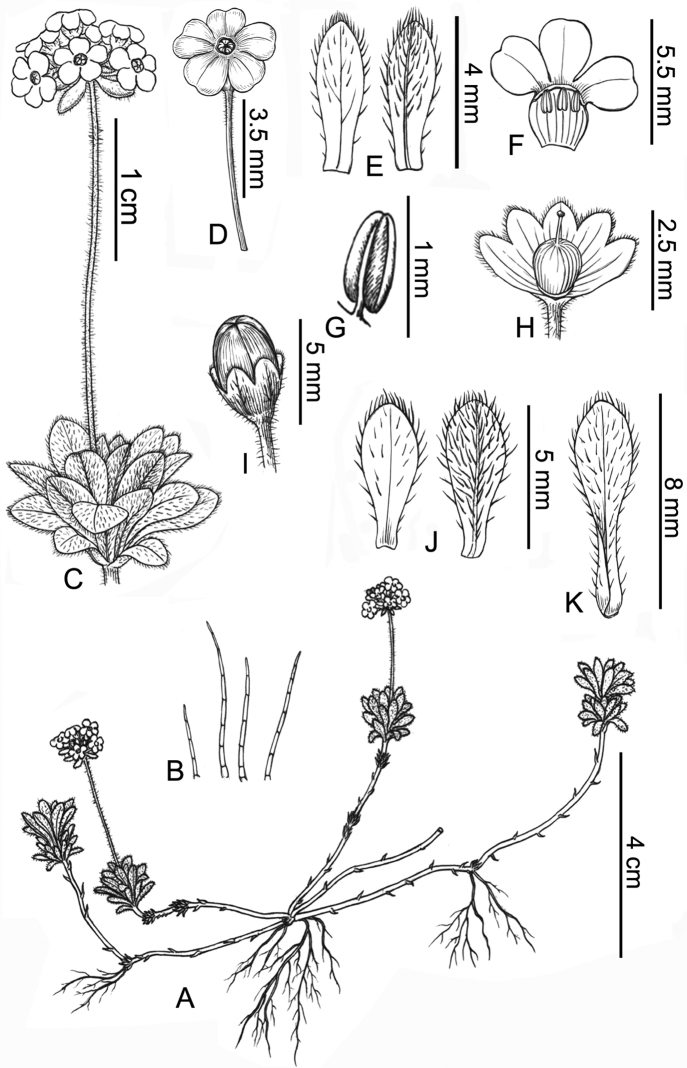
*Androsacechimingiana* sp. nov. **A** habit **B** multicellular hairs **C** leaf rosette on node and inflorescence **D** flower **E** bract on adaxial and abaxial surface **F** corolla (dissected) **G** anther **H** capsule with persistent calyx **I** capsule (split in valves) **J** outer leaf on adaxial and abaxial surface **K** inner leaf on abaxial surface. Drawn by Yun-Xiao Liu.

#### Conservation status.

The new species is narrowly distributed in Cangshan Mountain and the north part of Gaoligong Mountains with a limited number of populations. But each population includes numerous mature individuals and the habitat is usually intact. Therefore, the conservation status of the new species is assessed as Least Concern (LC) according to the guidelines for using the IUCN Red List categories and criteria (IUCN Standards and Petitions Subcommittee 2022).

**Figure 4.**
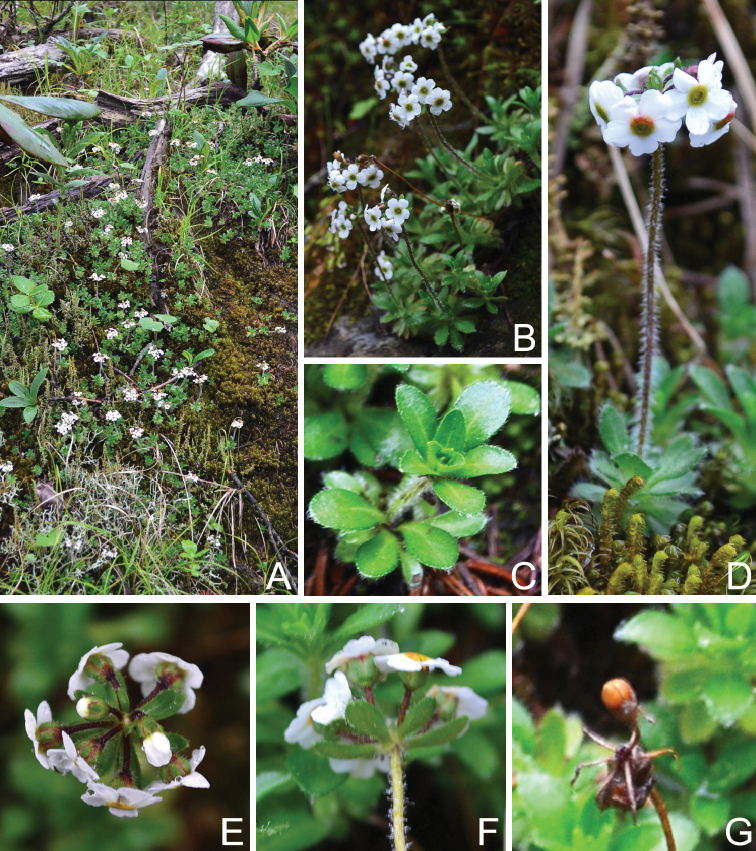
Living plant of *Androsacechimingiana* sp. nov. **A** habitat **B** habit **C** leaves **D** inflorescence **E** inflorescence showing calyx **F** inflorescence showing bract **G** capsule. Photographed by Yuan Xu.

#### Additional specimens examined (paratypes).

**China. Yunnan**: Dali City, Cangshan, *Alpine Garden Society Expedition to China 956* (LIV, K), *G. Forrest 7108* (E),*11678* (E), *1810* (E, K), *H.C. Wang 1236* (KUN barcode 0211513), *H.T. Tsai 53970* (E, KUN barcode 0211538, 0211539, 0211540, SZ barcode 00061069), *J.M. Delavay 1037* (E, P barcode P04907037, P01060625), *2800* (P barcode P05191797, P04623719, P04623720), *s.n.* (P barcode P01060637, P05191798), *K.L. Chu 228* (SZ barcode 00061070), *McLaren’s Collectors 95B* (P barcode P04553735), *R.C. Ching 25039* (KUN barcode 0211547, 0211548), *Sino-Amer. Bot. Exped. 1049* (E, KUN barcode 0211553, US barcode 03124154), *Zhongdian Exped. 63-3925* (KUN barcode 0211549, 0211550, 0211551); Gongshan Derung and Nu Autonomous County, Salwin-Kiukiang Divide, *H.R.E. Handel-Mazzetti 9280* (WU barcode WU0059689), *T.T. Yu 19385* (E, KUN barcode 0211515); Yangbi Yi Autonomous County, Diancangshan, *Sino-Amer. Bot. Exped. 539* (KUN barcode 0211552, US barcode 03124151).

#### Similar species and remarks.

The plants included in the paratype sheet (*H.R.E. Handel-Mazzetti 9280*, Fig. 2) of the new species are very small in size. Thus, the new species looks like *A.mollis*, but also can be easily distinguished by its obviously dimorphic leaves. The later collections from the type location and Cangshan also indicate that the leaves of new species are dimorphic and larger than *A.mollis*. Based on the result of our morphological observation, the new species is allied to *A.hookeriana* and *A.laxa*. All of them are laxly cespitose and both the morphology and the size of their inner leaves are similar. Handel-Mazzetti even identified the new species as *A.hookeriana* (e.g., signed on the sheet of *G. Forrest 7108* and *1810* E). But *A.hookeriana* is unique by its trimorphic leaves among these three species. The new species can also be easily distinguished from the other two species by its white flowers, obovate bracts, and campanulate calyx parted more than 1/2 of its length. The main morphological differences between the new species and its allies are listed in Table 1. In addition, the geographical isolation among these three species is significant. The new species is distributed in northwestern Yunnan, while *A.hookeriana* is distributed in the central Himalayas and *A.laxa* is distributed in Qinling-Daba Mountains.

**Table 1.** Main morphological differences among *Androsacechimingiana* and its allies.

Features	*A.chimingiana*	*A.laxa*	*A.hookeriana*	*A.mollis*
Leaves	Dimorphic	dimorphic	trimorphic	homomorphic, obovate-ligulate
Outer leaves	Obovate	spatulate to oblanceolate	lanceolate	n/a
Inner leaves	spatulate, petiole indistinct	elliptic to suborbicular, petiole narrowly winged	ovate-elliptic to suborbicular, petiole wingless	n/a
Bract	Obovate	lanceolate	linear	linear
Calyx	parted to 1/2 – ¾	parted to 1/2	parted less than 1/2	parted to less than 1/2
Corolla	White	pink	pink	Pink
Capsule	ellipsoidal	ellipsoidal	nearly spheroidal	nearly spheroidal

## Supplementary Material

XML Treatment for Androsace mollis

XML Treatment for Androsace chimingiana
